# Nociceptin/Orphanin FQ (N/OFQ) conjugated to ATTO594: a novel fluorescent probe for the N/OFQ (NOP) receptor

**DOI:** 10.1111/bph.14504

**Published:** 2018-11-06

**Authors:** M F Bird, R Guerrini, J M Willets, J P Thompson, G Caló, D G Lambert

**Affiliations:** ^1^ Department of Cardiovascular Sciences, Anaesthesia, Critical Care and Pain Management, Leicester Royal Infirmary University of Leicester Leicester UK; ^2^ Department of Chemical and Pharmaceutical Sciences and LTTA University of Ferrara Ferrara Italy; ^3^ Department of Molecular and Cell Biology University of Leicester Leicester UK; ^4^ Department of Medical Sciences, Section of Pharmacology and National Institute of Neuroscience University of Ferrara Ferrara Italy

## Abstract

**Background and Purpose:**

The nociceptin/orphanin FQ (N/OFQ) receptor (NOP) is a member of the opioid receptor family and is involved in a number of physiological responses, pain and immune regulation as examples. In this study, we conjugated a red fluorophore‐ATTO594 to the peptide ligand N/OFQ (N/OFQ_ATTO594_) for the NOP receptor and explored NOP receptor function at high (in recombinant systems) and low (on immune cells) expression.

**Experimental Approach:**

We assessed N/OFQ_ATTO594_ receptor binding, selectivity and functional activity in recombinant (CHO) cell lines. Live cell N/OFQ_ATTO594_ binding was measured in (i) HEK cells expressing NOP and NOP_GFP_ receptors, (ii) CHO cells expressing the hNOPGαqi5 chimera (to force coupling to measurable Ca^2+^ responses) and (iii) freshly isolated human polymorphonuclear cells (PMN).

**Key Results:**

N/OFQ_ATTO594_ bound to NOP receptor with nM affinity and high selectivity. N/OFQ_ATTO594_ activated NOP receptor by reducing cAMP formation and increasing Ca^2+^ levels in CHO_hNOPGαqi5_ cells. N/OFQ_ATTO594_ was also able to visualize NOP receptors at low expression levels on PMN cells. In NOP‐GFP‐tagged receptors, N/OFQ_ATTO594_ was used in a FRET protocol where GFP emission activated ATTO, visualizing ligand–receptor interaction. When the NOP_GFP_ receptor is activated by N/OFQ_ATTO594_, movement of ligand and receptor from the cell surface to the cytosol can be measured.

**Conclusions and Implications:**

In the absence of validated NOP receptor antibodies and issues surrounding the use of radiolabels (especially in low expression systems), these data indicate the utility of N/OFQ_ATTO594_ to study a wide range of N/OFQ‐driven cellular responses.

**Abbreviations**
DPN: diprenorphine
N/OFQ: nociceptin/orphanin FQ
NOP receptor: N/OFQ peptide receptor
PMN: polymorphonuclear cells
SB‐612111: 7‐[[4‐(2,6‐dichlorophenyl)‐1‐piperidinyl]methyl]‐6,7,8,9‐tetrahydro‐1‐methyl‐5*H*‐benzocyclohepten‐5‐ol hydrochloride

## Introduction

The nociceptin/orphanin FQ (N/OFQ) receptor (http://www.guidetopharmacology.org/GRAC/ObjectDisplayForward?objectId=320) is the newest member of the opioid receptor family (Lambert, 2008). It exhibits similar signalling mechanisms to the classical opioid receptors (http://www.guidetopharmacology.org/GRAC/ObjectDisplayForward?objectId=319, http://www.guidetopharmacology.org/GRAC/ObjectDisplayForward?objectId=317 and http://www.guidetopharmacology.org/GRAC/ObjectDisplayForward?objectId=318), through activation of a Gα_i_‐mediated G‐protein pathway (Meunier *et al*., 1995; Reinscheid *et al*., 1995). The NOP receptor, however, differs from the classical opioid receptors in a number of key areas. It has little to no affinity for the endogenous ligands of classical opioid receptors, nor does it have affinity for the opioid antagonist http://www.guidetopharmacology.org/GRAC/LigandDisplayForward?ligandId=1638. Furthermore, its own endogenous ligand, http://www.guidetopharmacology.org/GRAC/LigandDisplayForward?ligandId=1681, is highly selective for the NOP receptor, displaying little or no affinity for the classical opioid receptors.

Like the classical opioid receptors, NOP receptor expression has been demonstrated throughout the pain pathway, with antinociceptive actions ascribed to activation (Mollereau and Mouledous, 2000; Schroder *et al*., 2014; Ding *et al*., 2015). At initial identification, in mice and rat studies, it was believed that the NOP receptor had pronociceptive activity supraspinally; however, work by Ding and colleagues in non‐human primates demonstrated antinociceptive activity throughout the pain pathway underscoring important interspecies differences (Ding *et al*., 2015). The NOP receptor is expressed in a range of other non‐neuronal tissues and presumed levels vary widely (Lambert, 2008). NOP‐eGFP knock‐in mice have been used to examine expression‐function but with a neuronal focus (Ozawa *et al*., 2015). Outside the nervous system, we and others have shown the presence of the NOP receptor in immune tissues, but due to presumed ultra‐low expression, this has been suggested through PCR and modulation of immune function (Kruger *et al*., 2006; Zhang *et al*., 2013). While it is possible to obtain acceptable amounts of cerebral tissue to measure opioid expression through radioligand binding, the quantities of peripheral tissue available are most often inadequate for such studies.

Assessment of NOP receptor expression outside the brain is hindered by several key issues. The quantity of sample collected (e.g. immune cells) is often insufficient to perform radioligand assays, the current gold standard. Therefore, as noted above, receptor identity is often confirmed by either PCR or antibody methods. PCR detects mRNA, and these levels do not necessarily translate to functional protein (Guo *et al*., 2008). Antibodies for GPCRs have been shown to lack the high levels of specificity required to provide evidence of expressed protein (Michel *et al*., 2009). Previous work in our laboratory and others has demonstrated high levels of non‐selectivity using commercially available antibodies (Scherrer *et al*., 2009; Niwa *et al*., 2012). In standard Western blotting, we demonstrated the presence of bands at the expected weight in CHO cells expressing opioid receptors as well as those that had not been transfected with the opioid receptor of interest (Niwa *et al*., 2012). The importance of selectivity is highlighted in a study of δ (DOP) receptors (Scherrer *et al*., 2009). In their study identifying regions of expression of δ receptors, Scherrer and colleagues demonstrated large differences between areas of expression determined by antibody when compared to δ‐eGFP expressing mice (Scherrer *et al*., 2009).

Because the NOP receptor has no affinity for classical opioid ligands and N/OFQ is highly selective for NOP receptors, labelling of N/OFQ is an exciting possibility as a replacement for radioprobes and antibodies. Here, we describe for the first time a method using N/OFQ conjugated to the highly fluorescent ATTO dye (594 nm) and show this probe has a remarkable similarity to the unlabelled peptide.

## Methods

### Cell culture and immune cell preparation

Hams F12 was used to culture CHO cells expressing recombinant human opioid receptors (μ, δ and κ), DMEM/F12 1:1 media were used for both CHO_hNOP_ and CHO_hNOPGαqi5_ (from T Costa, Istituto Superiore di Sanità, Rome, Italy) and MEM was used for HEK cells expressing the recombinant NOP (HEK_hNOP_) receptor and HEK_hNOP‐GFP_. All media were supplemented with 10% FBS, 100 IU·mL^−1^ penicillin, 100 μg·mL^−1^ streptomycin and 2.5 μg·mL^−1^ fungizone. Cultures were maintained in selection media; for CHO cells containing the classical opioid receptors, 200 μg·mL^−1^ G418 was used. For HEK_hNOP_ cells, 200 μg·mL^−1^ hygromycin B was used. For both CHO_hNOP_ and CHO_hNOPGαqi5_ cells, 200 μg·mL^−1^ G418 and 200 μg·mL^−1^ hygromycin B were used. Recombinant cell lines were grown to confluency in T75 cm^3^ flasks in supplemented media at 37°C in 5%‐CO_2_/humidified air. Polymorphonuclear cells (PMN) were extracted from healthy volunteers as previously described (Thompson *et al*., 2013) and with University of Leicester volunteer research ethics committee approval. Up to 30 mL of blood was collected from each volunteer (mean age of 38; range 25–55, 4 male : 1 female) into Monovette blood collection tubes (Sardstedt, Germany) containing K‐EDTA (7.5 mL blood per tube, final EDTA concentration 1.6 mg·mL^−1^) and used within 1 h of venepuncture. PMN were extracted by centrifugation over an equal volume of Polymorphprep (Axis‐Shield, Dundee). Following extraction, PMN were cleared of any potential erythrocyte contamination by using a 1:1 dilution with BD PharmaLyse (Becton, Dickinson and Company, Oxford), resuspended in Krebs buffer (0.126 M NaCl, 2.5 mM KCl, 25 mM NaHCO_3_, 1.2 mM NaH_2_PO_4_, 1.2 mM MgCl_2_, 2.5 mM CaCl_2_) for counting and imaging. Extractions were carried out at room temperature, and the resulting cell suspension kept on ice until use (maximum 4 h). Viability and yields were quantified by Trypan Blue exclusion and counting using a haemocytometer (Strober, 2001).

### Radioligand binding

Radioligand binding was quantified in CHO cells stably transfected with μ, δ, κ and NOP receptors. Twenty to forty micrograms of membrane protein were incubated in 0.5 mL wash buffer (50 mM Tris–HCL, pH 7.4 KOH with the addition of 0.5% BSA), ∼0.8 nM http://www.guidetopharmacology.org/GRAC/LigandDisplayForward?ligandId=1612 (DPN; for classical opioid membranes) or ∼0.8 nM [^3^H]‐N/OFQ (for CHO_hNOP_ membranes), varying concentrations (1 pM–10 μM) of the control ligand or N/OFQ_ATTO594_. Non‐specific binding was measured in the presence of 10 μM naloxone (μ, δ, κ receptors) or 1 μM of N/OFQ (NOP receptor). Samples were incubated at room temperature for 1 h. Reactions were terminated by vacuum filtration onto polyethylenimine‐soaked Whatman GF/B filters, using a Brandel harvester (Bird *et al*., 2015).

### cAMP inhibition assay

HEK_hNOP‐GFP_ cells were suspended in Krebs/HEPES buffer, pH 7.4 NaOH containing http://www.guidetopharmacology.org/GRAC/LigandDisplayForward?ligandId=5190 (1 μM) and http://www.guidetopharmacology.org/GRAC/LigandDisplayForward?ligandId=388 (1 mM). Both the control ligand (unlabelled N/OFQ) and the N/OFQ_ATTO594_ were included in a range of concentrations (0.1 pM–1 μM) and incubated at 37°C for 15 min. Reactions were terminated through the initial addition of 20 μL 10 M HCl, followed by 20 μL 10 M NaOH and 200 μL 1 M Tris–HCl (pH 7.4) to equilibrate the pH. Reactions were centrifuged at 16 000× *g* and supernatant collected. The supernatant was incubated with http://www.guidetopharmacology.org/GRAC/LigandDisplayForward?ligandId=5096 and an in‐house prepared binding protein overnight at 4°C. Charcoal/BSA suspension was added and the reaction centrifuged at 16 000× *g*, following which, samples of the supernatant were counted as previously described (Kitayama *et al*., 2007).

### Confocal microscopy

At confluence, NOP expressing cells were passaged onto ethanol‐sterilized 28 mm Menzel glaser #1‐coverslips (Thermo Scientific, Loughborough, UK) and incubated for 24 h before use. Cells were perfused with Krebs buffer, pH 7.4 at 4°C using a temperature controller and microincubator (PDMI‐2 and TC202A; Burleigh, Digitimer, Cambridge, UK) when studying ligand–receptor binding, while in functional studies (internalization of the ligand–receptor complex), cells were incubated at 37°C. Where used, immune cells were plated onto coverslips pretreated with Celltak™ (1 μg·mL^−1^) (Sigma, UK) and incubated at 37°C for 1 h, before being washed in ice‐cold Krebs buffer.

N/OFQ_ATTO594_ was injected onto coverslips, allowing for a range of concentrations to be measured (1 pM−100 nM, cumulatively), following which, images were captured using a Nikon Eclipse C1Si microscope (Surrey, UK), using an oil immersion 60× objective. N/OFQ_ATTO594_ was allowed to incubate for 5 min at 4°C before the coverslip was washed with ice‐cold Krebs buffer. Following this incubation period, HEK_hNOP_ cells were imaged using the 594 nm wavelength laser, with images collected by the Nikon C1Si software. HEK_hNOP‐GFP_ and CHO_hNOPgαq/i5_ cells were imaged sequentially using the 594 nm wavelength followed by 488 nm to assess N/OFQ_ATTO594_ and GFP/Fluo4‐AM respectively, 10 s per frame. In order to determine specificity of binding, the NOP antagonist http://www.guidetopharmacology.org/GRAC/LigandDisplayForward?ligandId=1693 (1 μM) was pre‐incubated in desired cells for 15 min before addition of N/OFQ_ATTO594_. PMN were incubated with 100 nM N/OFQ_ATTO594_ and, following washing with ice‐cold Krebs, were imaged using the 594 nm wavelength laser. In all experiments, the laser and gain settings were maintained constant. For experiments with N/OFQ_ATTO594_ alone, settings were 37% 594 nm laser power, 7.10 gain‐red channel. In experiments including Fluo4‐AM stained cells (see below), 488 nm laser, an 18% power setting with 7.15 gain setting was used. Internalization studies were performed using 100 nM N/OFQ_ATTO594_ at 1 and 15 min time points (488 nm/594 nm wavelengths) in HEK_hNOP‐GFP_ cells. FRET studies were undertaken by measuring the binding of N/OFQ_ATTO594_ to HEK_hNOP‐GFP_. NOP‐GFP receptors were stimulated using a 488 nm laser, and measurements were made using the RED (594 nm) filter channel (Wallrabe *et al*., 2003). In order to confirm FRET‐pairing, several additional controls were performed (Snapp and Hegde, 2006). N/OFQ_ATTO594_ (100 nM) was incubated on HEK_hNOP_ cells (not expressing the GFP fluorophore) and measured using 488 nm laser. Furthermore, photobleaching of the ligand was performed, after binding to HEK_hNOP‐GFP_, by exposing it to 594 nm laser until fluorescence was undetectable, following which levels of GFP fluorescence intensity were measured. While GFP and ATTO594 may not be specifically designed as an optimum FRET pair, there is still significant crossover as demonstrated by spectra analyser data.

### CHO_hNOPGαqi5_ functional assays

In experiments using CHO_hNOPGαqi5_ cells (Camarda and Calo, 2013), the cells were incubated with 1 μM Fluo4‐AM for 45 min in Krebs buffer, following which they were washed for 3 min in 4°C Krebs *via* the perfusion system. A series of experiments were also performed at the physiological temperature of 37°C. N/OFQ_ATTO594_ (100 nM) was perfused while the cells were monitored under the confocal microscope [imaging using both 488 nm laser (Fluo4‐AM) and 594 nm laser (N/OFQ_ATTO594_)].

### Data analysis

All data are the mean of five experiments ± SEM as appropriate. Specimen confocal data sets are presented. All confocal images were analysed using ImageJ with resulting data analysed using GraphPad Prism‐v7. To measure corrected cell fluorescence, the formula, Corrected total cell fluorescence = Integrated density − (Area of selected cell × Mean fluorescence of background readings), was used to determine levels of N/OFQ_ATTO594_ as previously described (Burgess *et al*., 2010). In internalization studies, ImageJ tool plot profile was used to determine the position of N/OFQ_ATTO594_ and/or NOP_GFP_ relative to the cell membrane (Wallrabe *et al*., 2003). All experiments were performed unblinded.

### Materials

N/OFQ_ATTO594_, N/OFQ, dermorphin, Leu‐enkephalin and http://www.guidetopharmacology.org/GRAC/LigandDisplayForward?ligandId=1620 were synthesized in‐house at the University of Ferrara. Tritiated diprenorphine ([^3^H]‐DPN) and tritiated N/OFQ ([^3^H]‐N/OFQ) were purchased from Perkin Elmer (UK). Details of the synthesis of N/OFQ_ATTO594_ can be found in the Supporting Information Data S1 and Figures S1–S3.

### Nomenclature of targets and ligands

Key protein targets and ligands in this article are hyperlinked to corresponding entries in http://www.guidetopharmacology.org, the common portal for data from the IUPHAR/BPS Guide to PHARMACOLOGY (Harding *et al*., 2018), and are permanently archived in the Concise Guide to PHARMACOLOGY 2017/18 (Alexander *et al*., 2017).

## Results

### Binding selectivity and affinity

**Figure 1:**
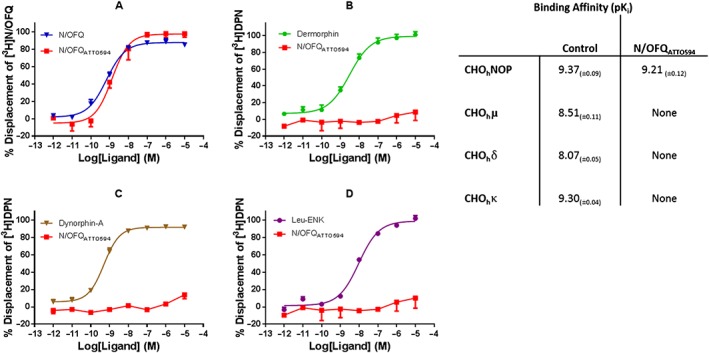
Displacement of [^3^H]‐N/OFQ in CHO_hNOP_ cell membranes by a range of concentrations of N/OFQ and N/OFQ_ATTO594_ (A). Displacement of [^3^H]‐DPN in CHO_hμ_ cell membranes by a range of concentrations of dermorphin and N/OFQ_ATTO594_ (B). Displacement of [^3^H]‐DPN in CHO_hκ_ cell membranes by a range of concentrations of dynorphin‐A and N/OFQ_ATTO594_ (C). Displacement of [^3^H]‐DPN in CHO_hδ_ cell membranes by a range of concentrations of Leu‐enkephalin and N/OFQ_ATTO594_ (D). Displacement binding affinities for N/OFQ_ATTO594_ and control ligands are summarised in the table (NOP: N/OFQ; μ: dermorphin; δ: Leu‐enkephalin; κ: dynorphin‐A). Data are the mean ± SEM of five experiments.

**Figure 2:**
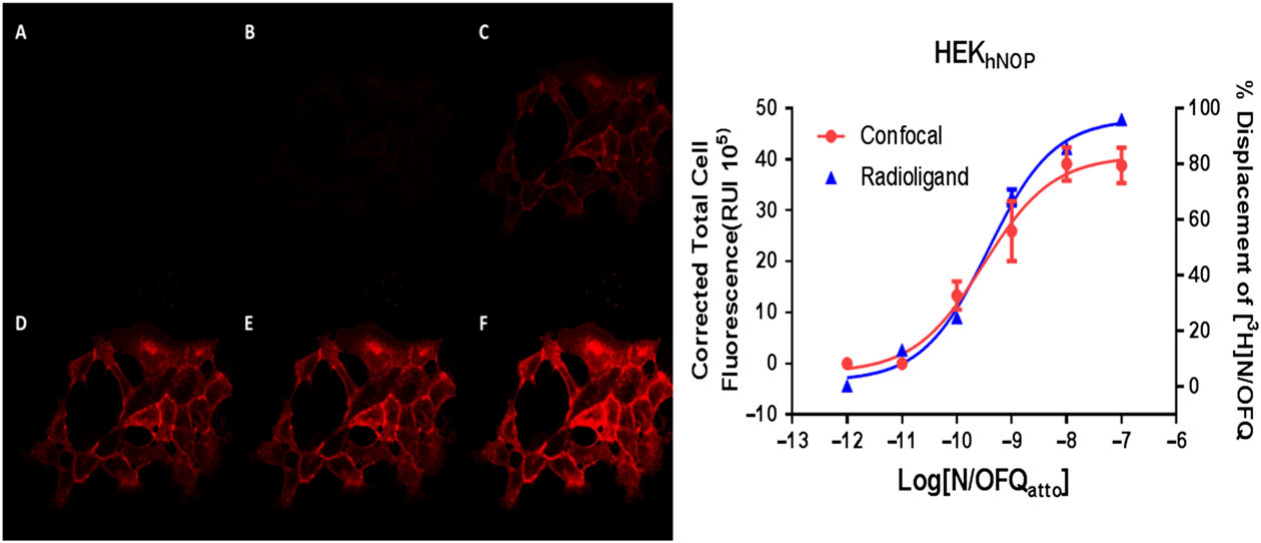
Concentration‐dependent binding of N/OFQ_ATTO594_ to HEK_hNOP_ cells using confocal microscopy and stimulation with 594 nm laser and [^3^H]‐N/OFQ. On the left representative images of the binding of various concentrations (A: 1 pM; B: 10 pM; C: 100 pM; D: 1 nM; E: 10 nM; F: 100 nM) of N/OFQ_ATTO594_ using confocal microscopy. On the right are the binding curves analysed as in methods overlayed with data from a [^3^H]‐N/OFQ displacement analysis; data are mean ± SEM for five experiments. Confocal experiments were performed at 4°C while radioligand binding assays were performed at room temperature.

**Figure 3:**
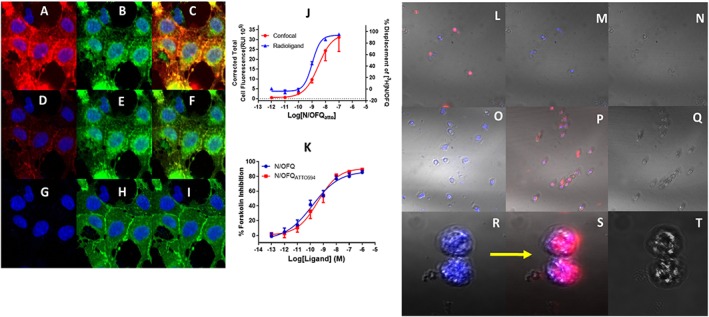
A–C: Binding of 100 nM N/OFQ_ATTO594_ in HEK_hNOP‐GFP_ split into the red channel (A) with N/OFQ_ATTO594_ binding, (B) the green channel to identify NOP_GFP_ tagged receptors and (C) a composite image demonstrating the interaction between N/OFQ_ATTO594_ and NOP_GFP_. (D–F) Binding of 1 nM of N/OFQ_ATTO594_ split into the red channel (D) showing N/OFQ_ATTO594_ binding, (E) the green channel identify NOP_GFP_ tagged receptors and (F) a composite image demonstrating overlapping binding of N/OFQ_ATTO594_ with NOP_GFP_. (G–I) Binding of 1 pM of N/OFQ_ATTO594_ showing split into the red channel (G) showing N/OFQ_ATTO594_ binding, (H) the green channel identify NOP_GFP_ tagged receptors and (I) a composite image demonstrating no binding of N/OFQ_ATTO594_ to NOP_GFP_. Nucleus is blue with DAPI stain. (J) Comparison between a radioligand displacement binding curve (using ^3^H‐N/OFQ at room temperature) and saturation curve measurement obtained from confocal microscopy. Data are mean ± SEM for five experiments. (K) Functional analysis of N/OFQ_atto_ activity (red) in a cAMP inhibition assay when compared to unlabelled N/OFQ. Data are mean ± SEM for five experiments performed at 37°C. (L) The ability of 100 nM N/OFQ_ATTO594_ to bind and visualize low expression systems is demonstrated in human PMN at 4°C. This binding can be disrupted by both unlabelled N/OFQ (1 μM) (M) and SB‐612111 (1 μM) (O). Furthermore, the classical opioid antagonist naloxone (1 μM) is unable to inhibit N/OFQ_ATTO594_ binding (P). In the enlarged image, unlabelled N/OFQ is pre‐incubated with PMN cells to occupy NOP receptors, before addition of 100 nM N/OFQ_ATTO594_ (R) [and as in M]), again inhibiting binding of the fluorescent ligand. After washing in Krebs to ‘clear’ the receptor for 10 min, 100 nM N/OFQ_ATTO594_ is able to bind to the surface of the PMN (S) [and as in L]). Bright field images for the accompanying experiments are shown in (N), (Q) and (T). All PNM data have been repeated for a total of five experiments. All confocal experiments were undertaken at 4°C.

Selectivity for N/OFQ_ATTO594_ was measured in CHO cells expressing human μ, δ, κ and NOP receptors. In CHO_hNOP_ cells, N/OFQ_ATTO594_ displaced [^3^H]‐N/OFQ in a concentration dependent and saturable manner (pK_i_: 9.21), which was not significantly (Student's *t*‐test) different from unlabelled N/OFQ (9.37) (Figure 1). N/OFQ_ATTO594_ failed to displace [^3^H]‐DPN in cells expressing μ, δ or κ receptors (Figure 1). In HEK_hNOP_ cells, N/OFQ_ATTO594_ also displaced [^3^H]‐N/OFQ in a concentration dependent and saturable manner (pK_i_ 9.14 ± 0.13; *n* = 5), which was not significantly (Student's *t*‐test) different from unlabelled N/OFQ (pK_i_: 9.34 ± 0.08; *n* = 5). A range of concentrations of N/OFQ_ATTO594_ were added to HEK cells expressing the NOP receptor and binding measured using 594 nm laser on a confocal microscope (Figure 2). N/OFQ_ATTO594_ bound in a concentration‐dependent and saturable manner, producing a pK_d_ of 9.51 ± 0.18 (*n* = 5; Figure 2). In HEK cells expressing GFP‐tagged NOP (HEK_hNOP‐GFP_) cells, N/OFQ_ATTO594_ bound to NOP with an affinity (pK_i_) of 8.98 ± 0.06 in displacement binding assays using [^3^H]‐N/OFQ as the radiolabel (Figure 3). When binding affinity was assessed directly using confocal microscopy, N/OFQ_ATTO594_ produced a binding affinity (pK_d_) of 8.53 ± 0.34, which was not significantly (Student's *t*‐test) different from that determined by displacement (Figure 3A–I, J). In confocal experiments, pre‐incubation with SB‐612111 abolished N/OFQ_ATTO594_ binding.

### Pharmacological activity

In order to determine whether conjugation of the fluorescent tag had any effect on functional activity, cAMP inhibition assays were performed (Figure 3K). The control ligand, unlabelled N/OFQ produced a pEC_50_ of 10.23 ± 0.25 and E_max_ of 85.43 ± 2.70% inhibition of forskolin‐stimulated cAMP formation. N/OFQ_ATTO594_ produced a pEC_50_ of 9.73 ± 0.29 and E_max_ of 89.30 ± 2.04%. There was no significant (Student's *t*‐test) difference in functional activity of N/OFQ_ATTO594_ when compared to N/OFQ (Figure 3K).

**Figure 4:**
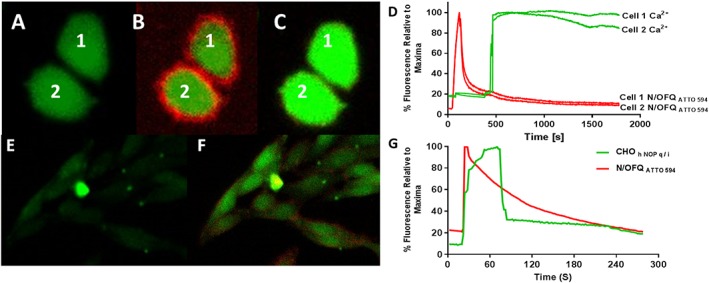
Live cell binding of N/OFQ_ATTO594_ and subsequent stimulation in CHO_hNOPGαqi5_ cells. Fluo4‐AM loaded CHO_hNOPGαqi5_ cells at 4°C (A) are incubated with 100 nM N/OFQ_ATTO594_ after 30 s (B) leading to release of calcium (cells labelled 1 and 2) (B and C). The process is demonstrated in the Supporting Information Video S1. This video has been created through ImageJ (eight frames per second), with N/OFQ_ATTO594_ added after 15 s, with the entirety of the video covering approximately 4.5 min. (D) A representative figure demonstrating N/OFQ_ATTO594_ binding to CHO_hNOPGαqi5_ cells at 4°C and the increase in Ca^2+^. Note: red is N/OFQ_ATTO594_ binding and green is Ca^2+^. Fluo4‐AM loaded CHO_hNOPGαqi5_ cells at 37°C (E) are incubated with 100 nM N/OFQ_ATTO594_ after 30 s, which leads to a prompt increase in binding and calcium (small field of cells; F). (G) A representative figure demonstrating binding of N/OFQ_ATTO594_ at 37°C and increase in Ca^2+^. Note: red is N/OFQ_ATTO594_ binding and green is Ca^2+^. These data are representative of *n* = 5.

In experiments performed at 4°C, 100 nM N/OFQ_ATTO594_ was added to CHO_hNOPGαqi5_ cells loaded with the calcium indicator, Fluo4‐AM (Figure 4A–D). An increase in red fluorescence can be seen around the membrane after addition of N/OFQ_ATTO594_, following which an increase in green fluorescence can be seen indicating activation of the G_αqi5_‐coupled NOP receptor and subsequent increase in cytosolic calcium (Figure 4A–D and Supporting Information Video S1). The kinetics of the rise in Ca^2+^ is slow as the experiment was performed at 4°C. Binding of N/OFQ_ATTO594_ at 37°C led to a more rapid increase of Ca^2+^ (Figure 4E–G).

### Low expression systems

Immune cells are known to express NOP mRNA and do not bind radiolabelled N/OFQ, but their function is modulated in response to N/OFQ. We next used N/OFQ_ATTO594_ to determine whether we could detect NOP at low levels of expression. Human PMN cells separated from healthy volunteers were seeded onto cover slips. At a temperature of 4°C, N/OFQ_ATTO594_ (100 nM) binding was observed (Figure 3L), indicating the presence of NOP receptors. Furthermore, pre‐incubation with unlabelled N/OFQ (1 μM; Figure 3M) or the selective NOP antagonist SB‐612111 (1 μM; Figure 3O) blocked the binding of N/OFQ_ATTO594_. The addition of naloxone (1 μM) had no effect on N/OFQ_ATTO594_ binding (Figure 3P); this is consistent with the selectivity of binding of N/OFQ_ATTO594_. In a further experiment to confirm NOP expression, PMN cells were pre‐incubated with N/OFQ before the addition of 100 nM N/OFQ_ATTO594_ (Figure 3R), demonstrating no binding of N/OFQ_atto594_. Cells were washed for several minutes in ice‐cold Krebs, following which 100 nM N N/OFQ_ATTO594_ was added for a second time. In this instance, binding was restored (Figure 3S). Brightfield images are shown in panels N, Q and T.

### NOP receptor internalization

**Figure 5:**
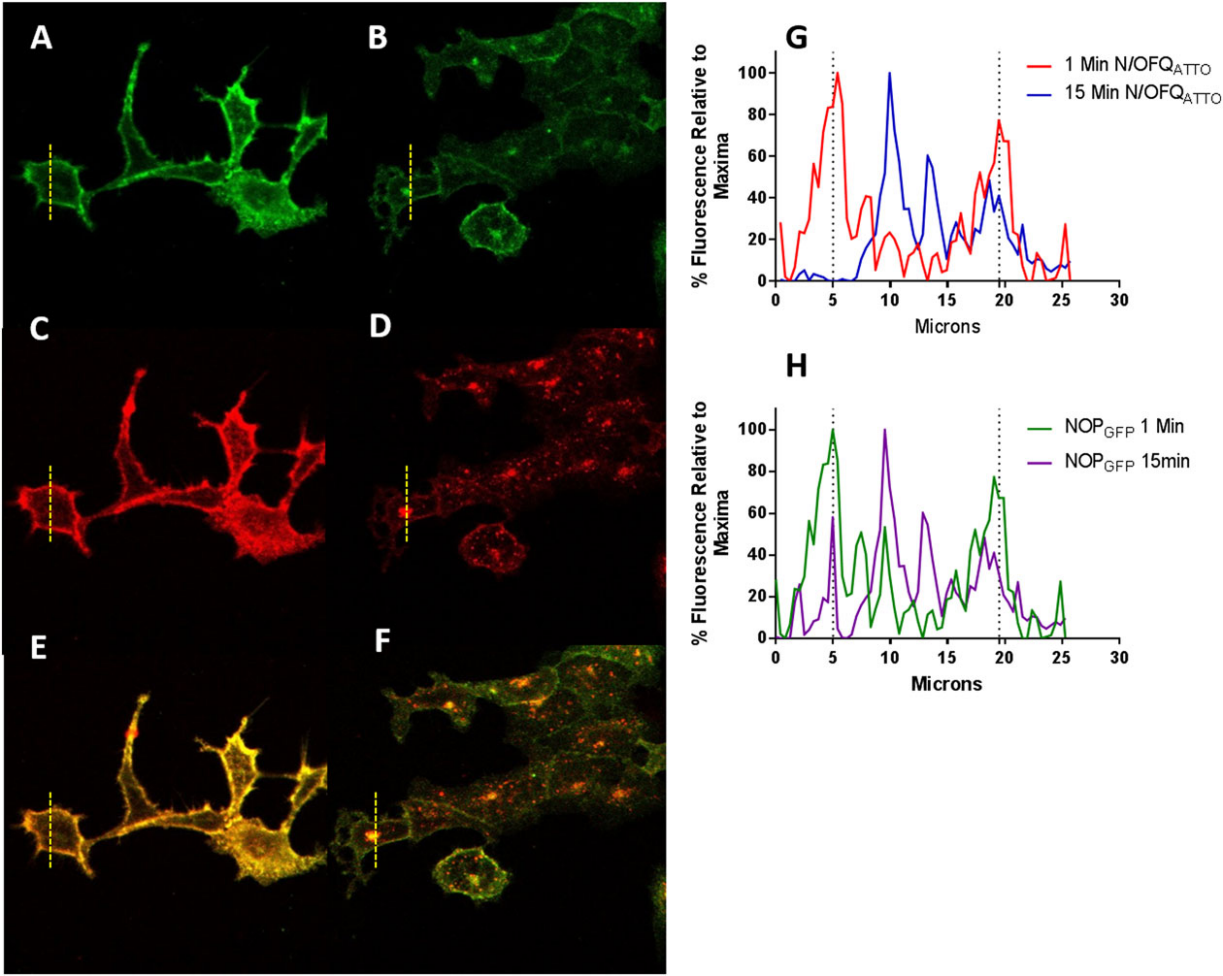
Use of N/OFQ_ATTO594_ and NOP_GFP_ to examine cell surface receptor expression. All images depict HEK_hNOP‐GFP_ cells. Panels A, C and E are at 4°C and measured 1 min after ligand addition while panels B, D and F are at 37°C, 15 min after ligand addition. Green channel for NOP_GFP_ is in A and B; red channel for N/OFQ_ATTO594_ is in C and D, and the overlap is in E and F. Panel G shows a representative graph of the initial binding of N/OFQ_ATTO594_ (red; 1 min after adding N/OFQ_ATTO594_) and subsequent change in localization (blue; 15 min after adding N/OFQ_ATTO594_). Panel H shows a representative graph of initial location of NOP_GFP_ (green; 1 min after adding N/OFQ_ATTO594_) and subsequent change in localization (purple; 15 min after adding N/OFQ_ATTO594_) In (G and H), the dotted lines are used to indicate the position of the membrane with the cell interior between the two.

We then used N/OFQ_ATTO594_ to track NOP receptor internalization. Binding was initially measured at 4°C using 100 nM of N/OFQ_ATTO594_ in HEK_hNOP‐GFP_ cells (Figure 5A, C and E) clearly demonstrating a localization of N/OFQ_ATTO594_ (Figure 5C) on the cellular membrane with GFP‐tagged NOP (A and overlay E). Following a temperature increase to 37°C, N/OFQ_ATTO594_ is seen to mobilize within the cell, away from the cell membrane (D). These pools of N/OFQ_ATTO594_ are shown to co‐localize with internalized NOP‐GFP (B and overlay F). Using ImageJ line analysis (dotted line in A–F), it is possible to assess the change in fluorescence at different time points across the cell after binding of N/OFQ_ATTO594_ (Figure 5G) and the subsequent localization of NOP‐GFP (Figure 5H).

**Figure 6:**
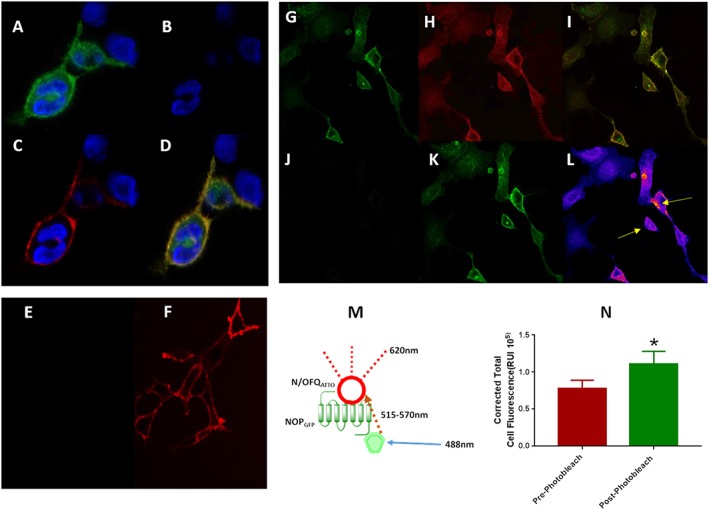
N/OFQ_ATTO594_ can be activated when in close proximity to GFP attached to the NOP receptor (FRET), as described in the cartoon in the middle of the figure (M). (A) Visualizes the NOP_‐GFP_ receptors stimulated by the 488 nm laser in the green filter window. (B) Demonstrates no ‘leak’ of fluorescence into the red window when using the green laser in the absence of N/OFQ_ATTO594_. Upon addition of 100 nM N/OFQ_ATTO594_, the ligand is stimulated through FRET pairing (C) to fluoresce when in close proximity to the NOP_GFP_ receptors. Signals are overlapped in (D). (E) In order to confirm FRET pairing, the 488 nm laser was used in conjunction with HEK_hNOP_ cells (no fluorescent linker present) to again demonstrate lack of activation of N/OFQ_ATTO594_. (F) Binding of N/OFQ_ATTO594_ was confirmed using 594 nm laser. Photobleaching of the acceptor molecule is a further method to confirm FRET pairing. HEK_hNOPGFP_ cells (G) were labelled with 100 nM N/OFQ_ATTO594_ (H) with binding of the receptor‐ligand complex shown as a composite image (I). N/OFQ_ATTO_ was exposed to 594 nm light until photobleaching was achieved (J) at which point changes in NOP_GFP_ fluorescence were measured (K) with the heatmap indicating increases of fluorescence shown and highlighted by arrows (blue: low and red: high) (L). (N) Average increase in NOP_GFP_ fluorescence after photobleaching of N/OFQ_ATTO594_; *P* < 0.05 Students t‐test. Data are the mean of eight experiments.

Because of the close proximity formed between ligand–receptor complexes (Figure 6M), a FRET stimulation was performed in HEK_hNOP‐GFP_ cells using N/OFQ_atto594_. When the receptor complex was formed, the 488 nm laser was used (Figure 6A–D), while filters in the red channel were viewed. In the absence of N/OFQ_ATTO594_, no image was obtained (Figure 6B). After administration of 100 nM N/OFQ_ATTO594_, a clear image demonstrating areas of interaction of NOP_GFP_‐N/OFQ_ATTO594_ was seen (Figure 6C), indicating FRET. The overlap of NOP_GFP_ and N/OFQ_ATTO594_ can be seen in an overlaid composite image (Figure 6D). In order to confirm FRET‐pairing, 100 nM N/OFQ_ATTO594_ was incubated with HEK_hNOP_ cells (not expressing GFP), following which detection of binding was attempted using the 488 nm wavelength laser (Figure 6E). N/OFQ_ATTO594_ was not stimulated by the laser in this environment. Binding of N/OFQ_ATTO594_ to the surface was confirmed by stimulation with 594 nm laser (Figure 6F). A second control experiment was performed, whereby 100 nM N/OFQ_ATTO594_ was incubated with HEK_hNOP‐GFP_ cells and exposed to prolonged stimulation through 594 nm laser wavelengths (Figure 6G–L). This produced photobleaching of the ligand (Figure 6J). The level of GFP fluorescence was taken before and after photobleaching and demonstrated that the corrected total cell fluorescence of GFP had a statistically significant increase from 0.78 ± 0.11 to 1.11 ± 0.17 (Student's *t*‐test; *P* < 0.05) (Figure 6N).

## Discussion

In this study, we report the synthesis and use of a novel fluorescent probe for the NOP receptor, N/OFQ_ATTO594_. We have conjugated ATTO594 to the highly selective endogenous NOP ligand N/OFQ, and this new ligand retains high NOP selectivity (over classical opioid receptors) and full agonist activity in (i) cAMP inhibition experiments performed in cells expressing NOP receptors (Kitayama *et al*., 2007), (ii) Ca^2+^ mobilisation experiments in cells co‐expressing NOP and chimeric G proteins (Camarda and Calo, 2013) and (iii) receptor internalization studies (Arttamangkul *et al*., 2000). N/OFQ_ATTO594_ was visibly detected over a range of concentrations when studied using confocal microscopy with sufficient sensitivity to determine a pK_d_; this did not differ from values obtained using the standard [^3^H]‐N/OFQ binding protocol. The nM affinity reported here, coupled with the high intrinsic brightness of ATTO594 allows for the visualization of NOP receptors in low expression systems, PMN. Use of cells expressing NOP receptors coupled to GFP allows further use of N/OFQ_ATTO594_ in a FRET‐based assay to document ligand–receptor interaction. Finally, this new ligand is well suited to studies of receptor internalization, and when coupled with a GFP tagged receptor, it should be possible to track both receptor and ligand fate following binding and activation and unbinding.

Because N/OFQ is an agonist for the NOP receptor, we have measured binding in confocal experiments at 4°C. At the more conventional 37°C, there would be substantial activation and loss of cell surface receptors (see below) as we have shown previously (Hashimoto *et al*., 2002; Barnes *et al*., 2007). The assessment of binding affinity would not be affected by temperature (Ahmadi *et al*., 2014) provided the ligand–receptor interaction was allowed to reach equilibrium, and we have a consistent assessment of affinity in HEK_NOP_ and HEK_hNOP‐GFP_. These values were not different from the native peptide, but coupling of NOP to GFP has reduced binding affinity. At 4°C, there is the potential for non‐equilibrium and an ideal solution would be to confirm affinity using association/dissociation time courses. In this paradigm, (i) the chamber floods with ‘free’ label making it impossible to determine bound ligand (i.e. separate from free) and (ii) experiments would be very long as the label is added cumulatively with the potential for internalization. We are reassured that our estimate of *K*_D_ is sensible as the values overlap with [^3^H]‐N/OFQ binding in membranes, and cognisant of the issues, we used two cell lines to confirm that all values are in good agreement. Measuring binding at the lower temperature followed by rapid warming allows the kinetics of internalization to be assessed. Despite measuring binding at 4°C, it is still possible to track receptor activation, at least at the level of Ca^2+^ in cells co‐expressing NOP and G_αqi5_ chimeric G‐protein. A similar shaped but more rapid response is also seen at 37°C. The kinetics at the lower temperature are substantially slower than we have previously reported for experiments performed at the higher temperature in population measurements, but the shape of the response is similar at all temperatures (Camarda and Calo, 2013).

Studies with GPCRs are, in general, hampered by lack of good antibodies for use in Western blotting (Hamdani and van der Velden, 2009; Jensen *et al*., 2009; Pradidarcheep *et al*., 2009; Berahovich *et al*., 2010; Cecyre *et al*., 2014; Talmont and Mouledous, 2014). This is likely due to the high degree of structural conservation both between families and more specifically in members of the same family, for example, the opioid family. Use of knockout tissue controls is often lacking, and as such, the validity of work using these probes could be questioned; this is not the case with our highly selective N/OFQ_ATTO594_. A more conventional strategy is to use radiolabelled probes. For NOP, this includes [^3^H]‐N/OFQ (McDonald *et al*., 2003), [^3^H]‐N/OFQ(1–13)‐NH_2_ (Hashiba *et al*., 2002), [^125^I]‐N/OFQ (Singh *et al*., 2013) and [^3^H]‐UFP‐101 [(Bird *et al*., 2016); their use is best employed where there is favourable access to tissue or where radioligand specific activity is high (e.g. with ^125^I). There are a series of elegant studies using radioligands for NOP in autoradiographic protocols (Slowe *et al*., 2001), but these retain the same issues regarding expression. Radioligands are in general not suited to use in tissues with presumed low expression, for example, vas deferens (Guerrini *et al*., 1998) and on immune cells (see below).

A further approach to track receptor fate is to use fluorescently labelled receptors, as we have done here with NOP_GFP_ receptors. An elegant example of how this can be used comes from the work of Evans *et al*. (2010). In this study, the authors produced fluorescently labelled isoforms of μ, δ and κ along with NOP receptors. Using a confocal paradigm, they went on to address receptor dimerization. They measured the degree of colour overlap in a manner similar to our data set examining overlap of GFP and ATTO to visualize ligand–receptor interaction either in the ‘conventional’ sense or in a FRET protocol. They conclusively showed that NOP was capable of hetero‐dimerization with all members of the opioid family (Evans *et al*., 2010).

In this study, as controls for FRET, we ensured that 488 nm wavelength alone did not stimulate ATTO (HEK_NOP_ cells without GFP tag) and in the presence of the GFP tag SB‐612111 (NOP antagonist) inhibits any FRET response. This indicates (i) ATTO is being stimulated by the emission spectra of GFP not through the laser, and (ii) it must be bound to the receptor to see the response, that is, locality which is the basis of FRET. Furthermore, photobleaching of the acceptor (N/OFQ_ATTO594_) leads to increased fluorescence measurements of GFP, a positive indicator of FRET pairing (Snapp and Hegde, 2006).

It has been known for many years that opioids are immune modulators (Vallejo *et al*., 2004), recently reviewed by Plein and Ritter (2018). The site of immune modulation is contentious, and we have reviewed this topic recently (Al‐Hashimi *et al*., 2013). Opioids could interact directly with the immune cell, modulate the activity of the hypothalamic–pituitary–adrenal axis and/or exert central actions involving glia (Hutchinson *et al*., 2011). The expression of opioid receptors on immune cells is the most contentious. There is well known modulation of immune cell activity (migration and cytokine release) (Liang *et al*., 2016), but we have failed to detect classical opioid (μ, δ and κ) receptor mRNA in any peripheral circulating immune cells (Al‐Hashimi *et al*., 2016). This is not consistent with the modulation of function, and in this area particularly, the lack of reliable antibodies for Western blot is a major disadvantage. It is possible that opioids could be working *via* a non‐opioid mechanism such as TLR4 receptors (Franchi *et al*., 2012), or there could be differences in circulating and resident immune cells. There is no evidence for the latter in the area of opioid pharmacology. What is clear is that all circulating immune cells that we have examined to date expressed mRNA for NOP receptors, and we and others have been able to report modulation of immune function (Singh *et al*., 2016). We have attempted to measure [^125^I]‐N/OFQ and [^3^H]‐N/OFQ binding to membranes from circulating mixed human immune cells (predominantly polymorphs). These experiments failed despite the use of relatively large amounts of membrane tissue, and we infer this is due to ultra‐low expression. After careful characterization in high expressing recombinant systems, in the present study, we went on to use N/OFQ_ATTO594_ in polymorphs from human volunteers and were able to detect binding. The small size of these immune cells (relative to the recombinants) and resolution of the microscope are limiting factors in pictorially demonstrating membrane location (see Supporting Information Figure S4). However, we were able to detect binding that could be blocked by pre‐occupying NOP with N/OFQ or the selective NOP antagonist SB‐612111 indicating both selectivity of binding to NOP and membrane location. Moreover, unlabelled N/OFQ could be effectively washed and replaced with N/OFQ_ATTO594_. These results demonstrate that NOP receptor mRNA measured in PCR experiments is effectively translated into protein capable of binding N/OFQ and, unlike classical opioid receptors, provides a target to explain the observed immune modulation.

Upon activation opioid receptors are internalized in an arrestin‐driven fashion (Williams *et al*., 2013). This is a fairly standard mechanism utilized by a number of GPCRs (Peterson and Luttrell, 2017) and leads to reduced cellular responsiveness or desensitization. For the NOP receptor, we have used a BRET protocol (using Renilla luciferase‐NOP and Renilla GFP on arrestin) to show efficient arrestin coupling (Malfacini *et al*., 2015). We have used radioligand binding to measure loss of cell surface NOP receptors following a desensitizing challenge. These latter studies were in high expressing (Hashimoto *et al*., 2002) or inducible expressing (Barnes *et al*., 2007) systems. The issues with these types of experiments is complete removal of the desensitizing challenge as any residual receptor occupancy would effectively reduce apparent density leading to an erroneous conclusion as to loss of receptors. The fluorescent probe we have designed, as an agonist itself, overcomes these problems. In this study, we show that receptors on the cell surface (GFP tagged) with N/OFQ (ATTO) bound leave the cell surface together (line analysis); ligand and receptor appear to co‐localize inside the cell. Use of N/OFQ_ATTO594_ to study internalization and the role of arrestins is particularly apposite here as there is now compelling evidence to suggest that agonists biased away from arrestin recruitment produce analgesia without the development of tolerance, a game changer in the development of analgesics for chronic pain.

Moreover, it is also possible to measure the ligand receptor interaction process in a FRET‐based assay coupling the ATTO‐labelled peptide with a GFP‐labelled receptor in cell lines. This will also be possible in NOP‐eGFP knock‐in mice (Ozawa *et al*., 2015).

In summary, we describe a novel ligand for use in the study of live‐cell ligand–receptor interaction and tracking movement of liganded cell surface receptors. Use of higher resolution confocal technologies will facilitate a more detailed study of the ‘unbinding’ and recycling process. Interesting further possibilities for this new ligand include its use in (i) brain sections and (ii) receptor binding protocols where the fluorescent label totally replaces the radioligand; this would revolutionize binding methodology for this receptor and potentially other members of the opioid family.

## Author contributions

M.F.B. collected and analysed the data and wrote the paper. R.G. designed and synthesised the ligand and wrote the paper. J.M.W. and J.P.T. wrote the paper and obtained funding. G.C. designed the ligand and wrote the paper. D.G.L. analysed the data, wrote the paper and obtained funding.

## Conflict of interest

The authors declare no conflicts of interest.

## Declaration of transparency and scientific rigour

This http://onlinelibrary.wiley.com/doi/10.1111/bph.13405/abstract acknowledges that this paper adheres to the principles for transparent reporting and scientific rigour of preclinical research recommended by funding agencies, publishers and other organisations engaged with supporting research.

## Supporting information


**Data S1** Chemistry.
**Figure S1** Analytical HPLC profile of the [Cys(ATTO 594)^18^]N/OFQ‐NH_2_ reaction mixture.
**Figure S2** Analytical HPLC profile of the purified [Cys(ATTO 594)^18^]N/OFQ‐NH_2_.
**Figure S3** Mass spectrum of the purified [Cys(ATTO 594)^18^]N/OFQ‐NH_2_.
**Figure S4** A limited image z‐series stack for 100 nM N/OFQ_ATTO594_ binding to PMN is presented, panel A. In panel B, representative ‘slices’ at top, middle and bottom are shown.

**Video S1** N/OFQ_ATTO594_ binding and increased Ca^2+^ at 4°C.
