# Supplementary material for: Nociceptin/Orphanin FQ (N/OFQ) conjugated to ATTO594: a novel fluorescent probe for the N/OFQ (NOP) receptor
Source: Br J Pharmacol. 2018 Nov 6;175(24):4496–506. doi: 10.1111/bph.14504 (PMC6255954; doi:10.1111/bph.14504)

Supplementary data

1. Chemistry

Synthesis of [Cys(ATTO 594)^{18}]N/OFQ-NH_2. The conjugation of [Cys^{18}]N/OFQ-NH_2 to ATTO 594 (purchased from ATTO-TEC GmbH Am Eichenhang 50 D-57076 Siegen Germany, structure not available) was achieved using the classic thiol-Michael reaction. A solution of maleimide derivative fluorescent probe (1 mg, 1 equiv.) in CH_3CN (250 µL) was added to a stirred solution of [Cys^{18}]N/OFQ-NH_2 (1.1 equiv.) in 250 µL of H2O, followed by the addition of 25 µL of $NaHCO_3$ 5%. The mixture was stirred in the dark under a nitrogen atmosphere and at room temperature for 15 minutes. The reaction was monitored by analytical HPLC (Figure S1) and MS analysis. After completion of the reaction, preparative HPLC purification of the reaction mixture gave the desired final product in quantitative yield (see Figure S2 and S3 for HPLC and MS of the final product, respectively).

2. Binding of N/OFQ$_{ATTO594}$ is to the exterior of the cell

Figure S4 is a z-series stack for a pair of PMN cells. The cells are small and round presenting problems with microscope resolution. In the main paper pre-incubation with unlabelled N/OFQ and the NOP antagonist SB612111 completely prevented the binding of N/OFQ$_{ATTO594}$ indicating the resulting label as shown both in the main paper and Figure S4 is on the external surface of the cell.

3. Figure legends

Figure S1: Analytical HPLC profile of the [Cys(ATTO 594)^{18}]N/OFQ-NH_2 reaction mixture.

Figure S2: Analytical HPLC profile of the purified [Cys(ATTO 594)^{18}]N/OFQ-NH_2.

Figure S3: Mass spectrum of the purified [Cys(ATTO 594)^{18}]N/OFQ-NH_2.

Figure S4: A limited image z-series stack for 100nM N/OFQ$_{ATTO594}$ binding to PMN is presented panel A. In panel B representative ‘slices’ at top, middle and bottom are shown.

Figure S1:

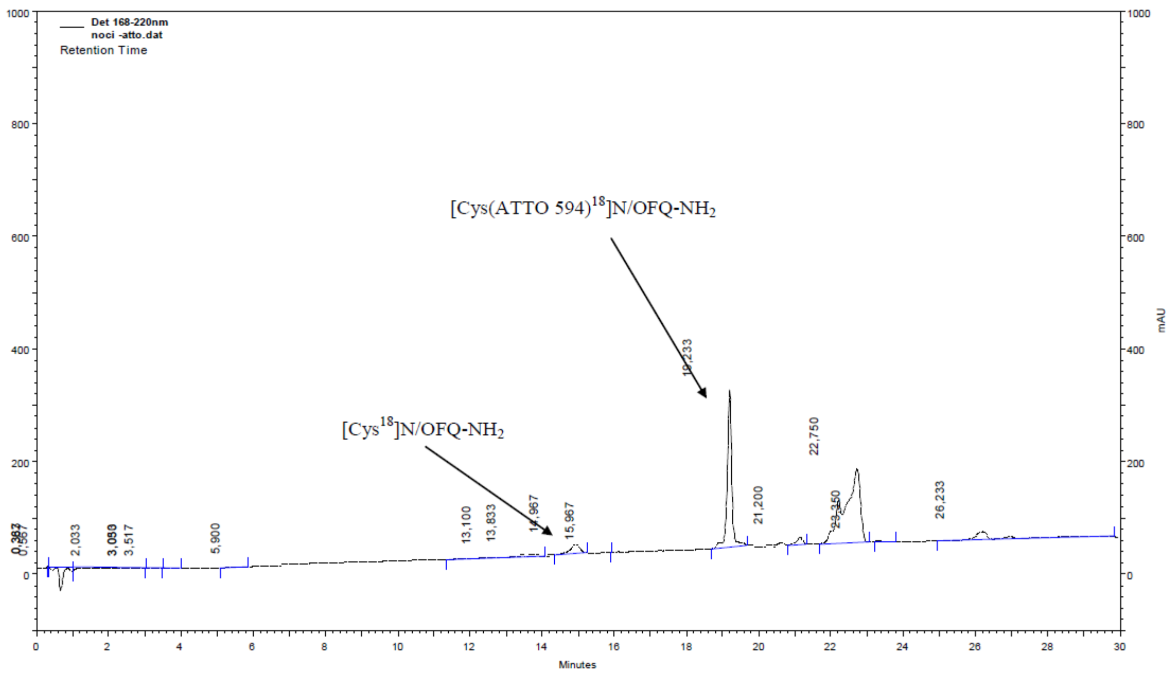

Figure S2:

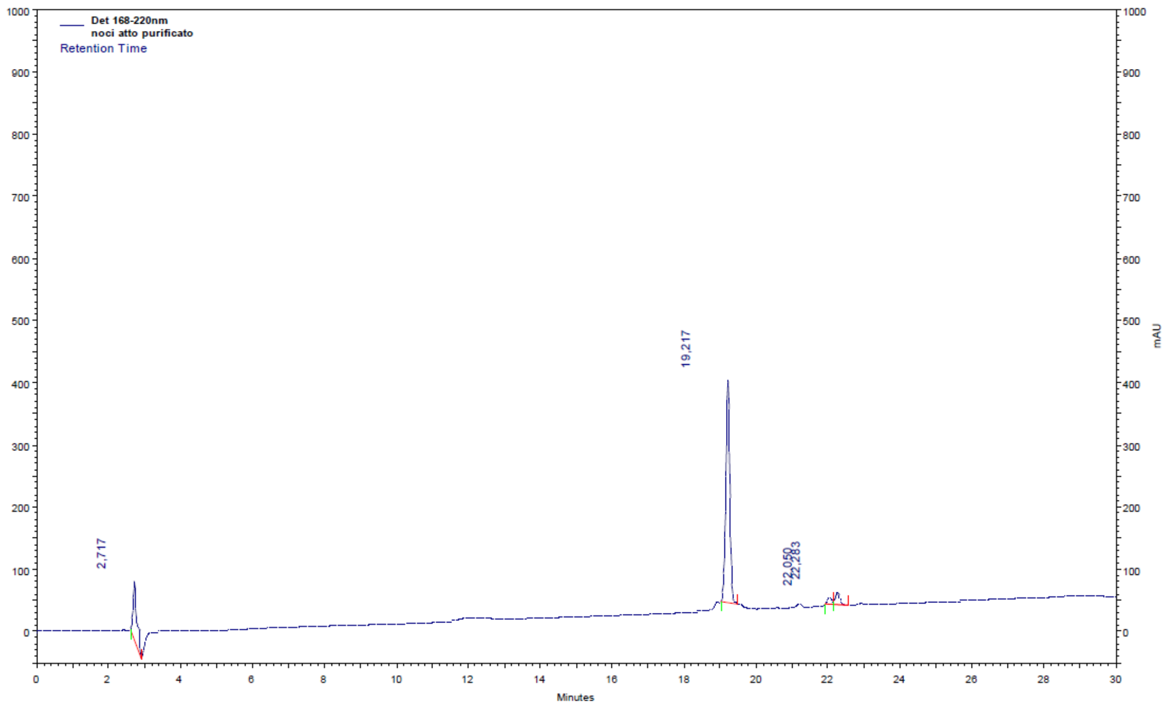

Figure S3:

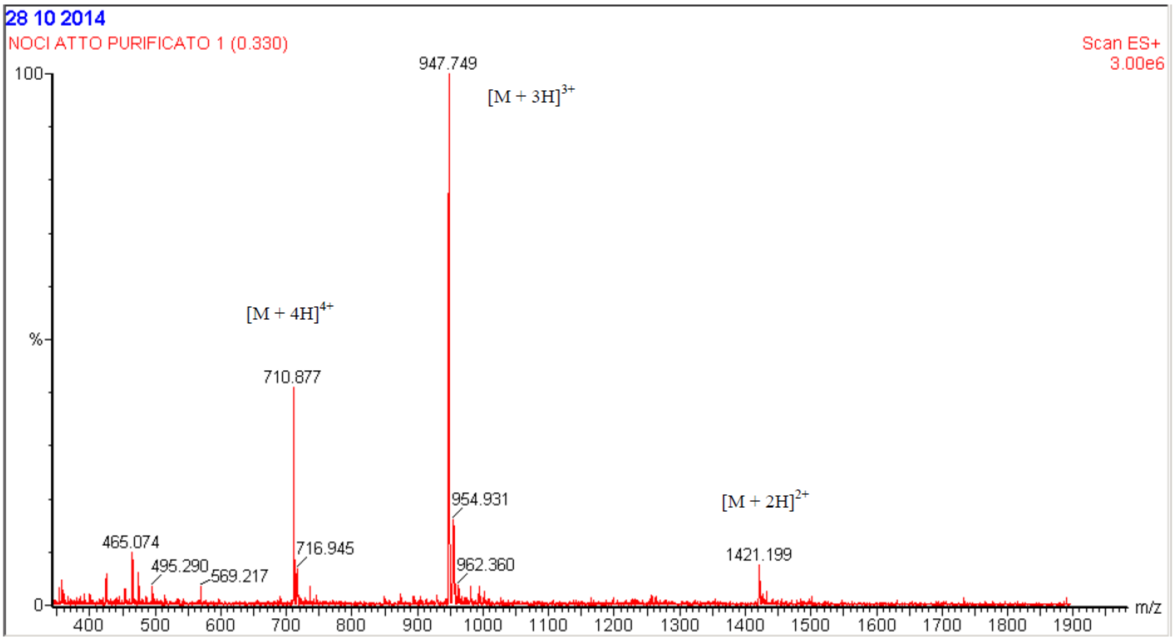

Figure S4:

Sum of Z-stack First Slice 1/179 Middle slice 89/179 Middle slice 111/179 End slice 170/179

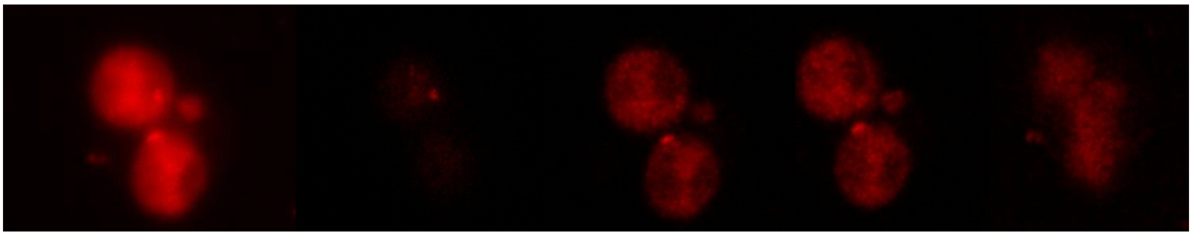

Supplement: Supplementary file 1 — Data S1 Chemistry. Figure S1 Analytical HPLC profile of the [Cys(ATTO 594)18]N/OFQ‐NH2 reaction mixture. Figure S2 Analytical HPLC profile of the purified [Cys(ATTO 594)18]N/OFQ‐NH2. Figure S3 Mass spectrum of the purified [Cys(ATTO 594)18]N/OFQ‐NH2. Figure S4 A limited image z‐series stack for 100 nM N/OFQATTO594 binding to PMN is presented, panel A. In panel B, representative ‘slices’ at top, middle and bottom are shown. [file BPH-175-4496-s001.pdf]
